# Diagnostic pathways in returning travellers: integrating Ebola outbreak screening into comprehensive workup

**DOI:** 10.1016/j.nmni.2026.101787

**Published:** 2026-06-11

**Authors:** Alberto Rizzo, Federico Fama, Davide Mileto, Marta Colaneri, Andrea Gori, Alberto Dolci

**Affiliations:** a Laboratory of Clinical Microbiology, Virology and Bioemergencies, Luigi Sacco Hospital, Regional Center for Infectious Diseases (CEREMI), Milan, Lombardy Region, Italy; b Department of Infectious Disease, Luigi Sacco Hospital, Regional Center for Infectious Diseases (CEREMI), Milan, Lombardy Region, Italy; c Department of Biomedical and Clinical Sciences (DIBIC), University of Milan, Milan, Italy

Dear Editor,

The outbreak of Ebola disease caused by Bundibugyo virus (BDBV) in the Democratic Republic of the Congo and Uganda is inevitably going to cause a rise of febrile returning travellers to reference centres in non-endemic countries [1]. While exposure assessment, clinical evaluation, isolation, public-health notification, and BDBV testing within appropriate containment are established; subsequent investigation is less defined. The current BDBV outbreak highlights the need to move from a pathogen-centred preparedness model to a traveller-centred diagnostic pathway. Although potential exposure to BDBV appropriately triggers referral, isolation, and specialised testing, most evaluated patients will ultimately receive an alternative diagnosis. The clinical challenge therefore extends beyond exclusion of BDBV itself, encompassing timely recognition of other infectious causes of febrile illness in returning travellers.

Excluding BDBV or any viral haemorrhagic fever (VHF) does not close the diagnostic workup. In fact, early BDBV symptoms are shared with malaria, arboviral infection, enteric fever, and respiratory infections. The exclusion of VHF justifies de-escalation of precautions, but not of diagnostic effort: missing a treatable infection or a surveillance-relevant pathogen remains a real risk.

The imported influenza A(H9N2) case reported in Italy after travel is instructive, although from a BDBV not-affected country. An unsubtypable influenza A result led to reference-laboratory confirmation, sequencing, antiviral treatment, public-health notification, and contact tracing [2]. Thus, excluding the most feared diagnosis like seasonal influenza does not complete assessment of a febrile returning traveller.

Multiplex syndromic panels have an important role both when BDBV is suspected and when it has been excluded—covering returning individuals from affected areas. When initiated in parallel with BDBV testing, they reduce diagnostic delay; when an alternative aetiology is confirmed alongside a negative BDBV result, they raise the post-test probability of genuine BDBV exclusion. Speed remains essential: diagnoses must be available rapidly enough to inform clinical and public-health actions. Preparedness should not create a diagnostic bottleneck whereby exclusion of a rare high-consequence pathogen delays recognition of more common, treatable, or epidemiologically relevant infections. Parallel diagnostic pathways may help overcome this limitation.

In a Sierra Leone Ebola treatment unit, non-Ebola diagnoses were frequent and rapid diagnostics supported clinical management [3]. Studies in febrile travellers suggest that multiplex panels can identify the most involved pathogens, although performance varies and traditional methods remain necessary [4,5].

Reference centres would benefit from adopting staged diagnostic algorithms rather than isolated BDBV testing. Such algorithms should specify which specimens can be processed at each biosafety level and after which inactivation or containment steps. The implications extend beyond the current BDBV outbreak. Similar diagnostic challenges arise whenever travellers are evaluated for other high-consequence infections, including other VHFs such as Lassa fever, Marburg virus disease, or Crimean-Congo haemorrhagic fever. In all these scenarios, the initial need for strict containment should be integrated with a structured diagnostic strategy capable of rapidly identifying alternative causes of illness.

Table 1 presents pragmatic approaches of syndromic testing stratified by clinical presentation, to be integrated with epidemiological features.

**Table 1:** Syndromic testing approach after initial VHF assessment in symptomatic returning travellers referred to reference centres. Abbreviations: BDBV=Bundibugyo virus; COVID-19 = Coronavirus disease 2019; NAAT = nucleic acid amplification test; RSV = respiratory syncytial virus; VHF = viral haemorrhagic fever.

Clinical presentation after initial BDBV assessment	Diagnostic approach to consider	Examples of diagnoses	Why it matters
Fever, headache, myalgia	Malaria rapid diagnostic test; blood-based multiplex panel for tropical fevers	Malaria, dengue, chikungunya	Time-sensitive treatment; epidemiological tracking
Fever, headache, myalgia; mild-moderate respiratory symptoms	Respiratory multiplex NAAT; influenza A subtyping if unsuitable for seasonal classification or zoonotic exposure is suspected	Influenza, RSV, COVID-19, non-seasonal Influenza A subtypes	Antiviral therapy options; outbreak surveillance
Diarrhoea or vomiting	Gastrointestinal multiplex panel	Viral, bacterial, and protozoal gastroenteritis	Supportive care guidance; outbreak investigation
Pulmonary infiltrates, chronic symptoms	Mycobacterial screening; respiratory pathogens panel	Tuberculosis, avian influenza	Prolonged treatment initiation; urgent public-health action

This approach broadens diagnostic scope without compromising VHF precautions. Multiplex syndromic panels, initiated in parallel with VHF testing and continued after exclusion, serve a dual purpose: they accelerate diagnosis and strengthen diagnostic confidence. The framework applies whenever a VHF is suspected and subsequently excluded, and the diagnostic investigation continues.

Dedicated referral centres combining bioemergency facilities, infectious-disease emergency care, and advanced microbiology laboratories are uniquely positioned to implement such integrated pathways. In these settings, containment procedures and comprehensive syndromic diagnostics can proceed simultaneously rather than sequentially, reducing delays in both patient management and public-health response.

Reference centres with established bioemergency expertise are well positioned to lead this effort, and to demonstrate that comprehensive diagnostics and rigorous containment are not competing priorities, but complementary components of modern preparedness. Diagnostic de-escalation after VHF exclusion should not be interpreted as diagnostic closure; rather, it should mark the transition toward a broader investigation of febrile illness in the returning traveller.

## CRediT authorship contribution statement

**Alberto Rizzo:** Conceptualization, Writing – original draft, Writing – review & editing. **Federico Fama:** Writing – original draft, Writing – review & editing. **Davide Mileto:** Project administration, Writing – review & editing. **Marta Colaneri:** Project administration, Writing – review & editing. **Andrea Gori:** Supervision, Writing – review & editing. **Alberto Dolci:** Supervision, Writing – review & editing.

## Declaration of competing interest

Authors declare no competing interests.
